# Virtual biopsy in abdominal pathology: where do we stand?

**DOI:** 10.1259/bjro.20220055

**Published:** 2023-02-28

**Authors:** Arianna Defeudis, Jovana Panic, Giulia Nicoletti, Simone Mazzetti, Valentina Giannini, Daniele Regge

**Affiliations:** Department of Radiology, Candiolo Cancer Institute, FPO-IRCCS, Strada Provinciale 142, Candiolo (TO), Piedmont, Italy; Department of Surgical Sciences, University of Turin, Via Verdi 8,Turin, Piedmont, Italy; Department of Surgical Sciences, University of Turin, Via Verdi 8,Turin, Piedmont, Italy; Department of Electronics and Telecommunications, Polytechnic of Turin, Corso Duca degli Abruzzi 24, Turin, Piedmont, Italy; Department of Surgical Sciences, University of Turin, Via Verdi 8,Turin, Piedmont, Italy; Department of Electronics and Telecommunications, Polytechnic of Turin, Corso Duca degli Abruzzi 24, Turin, Piedmont, Italy; Department of Radiology, Candiolo Cancer Institute, FPO-IRCCS, Strada Provinciale 142, Candiolo (TO), Piedmont, Italy; Department of Surgical Sciences, University of Turin, Via Verdi 8,Turin, Piedmont, Italy; Department of Radiology, Candiolo Cancer Institute, FPO-IRCCS, Strada Provinciale 142, Candiolo (TO), Piedmont, Italy; Department of Surgical Sciences, University of Turin, Via Verdi 8,Turin, Piedmont, Italy; Department of Radiology, Candiolo Cancer Institute, FPO-IRCCS, Strada Provinciale 142, Candiolo (TO), Piedmont, Italy; Department of Surgical Sciences, University of Turin, Via Verdi 8,Turin, Piedmont, Italy

## Abstract

In recent years, researchers have explored new ways to obtain information from pathological tissues, also exploring non-invasive techniques, such as virtual biopsy (VB). VB can be defined as a test that provides promising outcomes compared to traditional biopsy by extracting quantitative information from radiological images not accessible through traditional visual inspection. Data are processed in such a way that they can be correlated with the patient’s phenotypic expression, or with molecular patterns and mutations, creating a bridge between traditional radiology, pathology, genomics, and artificial intelligence (AI). Radiomics is the backbone of VB, since it allows the extraction and selection of features from radiological images, feeding them into AI models in order to derive lesions' pathological characteristics and molecular status. Presently, the output of VB provides only a gross approximation of the findings of tissue biopsy. However, in the future, with the improvement of imaging resolution and processing techniques, VB could partially substitute the classical surgical or percutaneous biopsy, with the advantage of being non-invasive, comprehensive, accounting for lesion heterogeneity, and low cost. In this review, we investigate the concept of VB in abdominal pathology, focusing on its pipeline development and potential benefits.

## Introduction

In abdominal pathology, diagnosis is usually obtained by combining information from physical examination, laboratory tests, imaging, and tissue biopsy. The latter plays a key role in detecting, characterising, and providing molecular information from the extracted tissues. Unfortunately, due to tumour heterogeneity, the retrieved specimens usually do not fully represent the lesion.^1^ Moreover, biopsy presents several drawbacks including the risk of adverse events, such as bleeding, physical, and psychological discomfort.^2^

**Figure 1.**
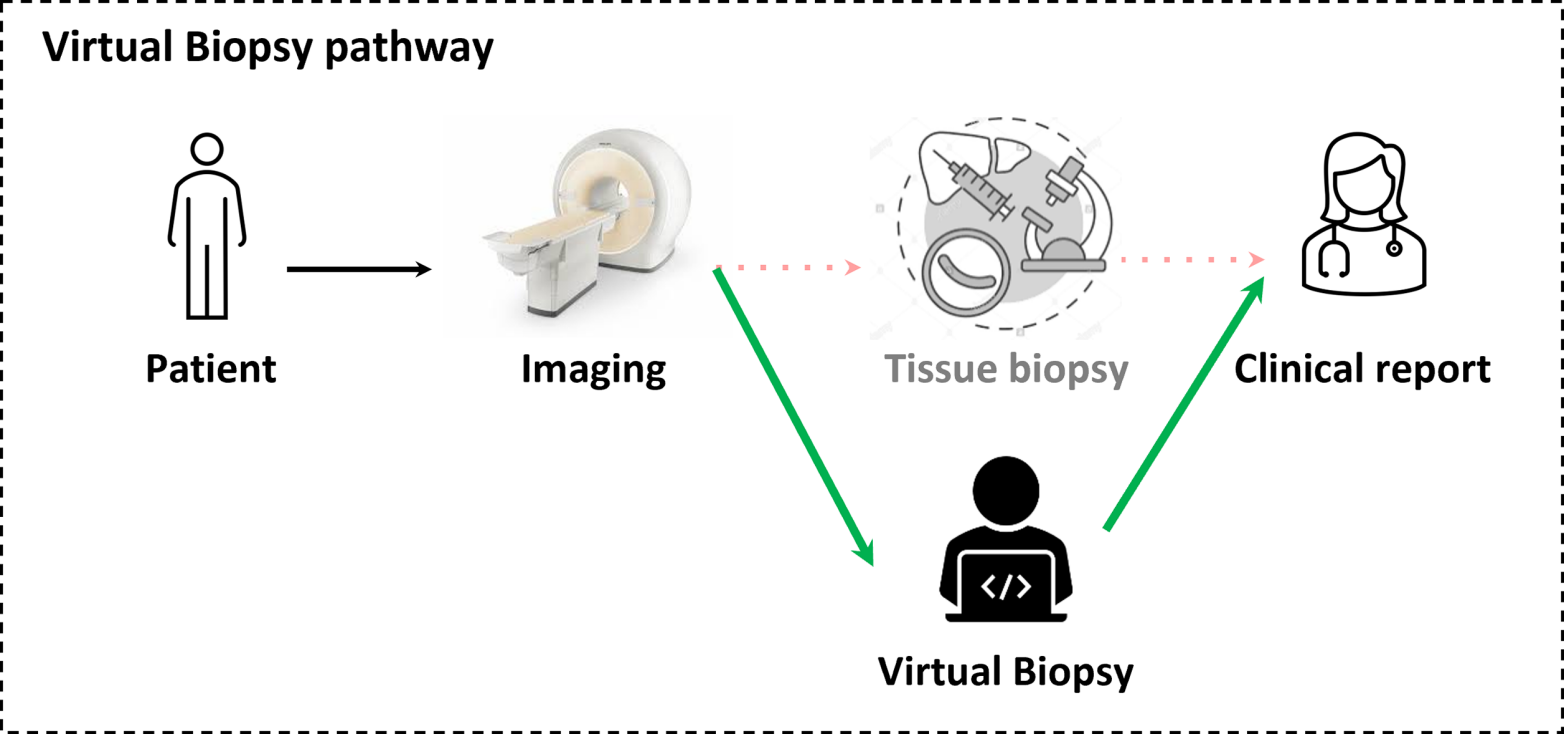
Scheme of the proposed virtual biopsy pathway. The patient undergoes the MRI imaging process, then the virtual biopsy will be performed instead of a traditional tissue biopsy. Finally, according to the results, the radiologist will write the clinical report processed image.

The advent of anatomic and functional diagnostic imaging has improved the non-invasive characterisation of tissues and brought a decrease in the overall number of biopsies.^3^ However, considerable limitations still exist, because imaging mainly provides a subjective evaluation that is strongly influenced by reader experience and that retains a variable degree of uncertainty. Contextually, new technologies are unveiling the molecular complexity of the disease, in particular cancer, through the analysis of tissue specimens. Therefore, there is an emerging need to provide deeper insight into the molecular drivers of disease through imaging. This paradigmatic shift is encompassed in the definition of virtual biopsy (VB), a test that provides promising outcomes compared to traditional biopsy by extracting quantitative information from radiological images not accessible through visual inspection. Data are processed to deliver information on the patient’s phenotypic expression, or molecular drivers of disease, creating a bridge between traditional radiology, pathology, genomics, and artificial intelligence (AI). The information extracted by VB is complementary to that obtained through traditional visual inspection of radiologists, since it is imperceptible to the human eye.^4^ In the future, VB could partially substitute traditional biopsy and have the following potential benefits (Figure 1): complication risks of VB are null; costs are low since the information is extracted from readily available radiological images; the report can be immediately provided to the patient, affecting positively the treatment timetable.^1^

VB biomarkers are mainly obtained with radiomics, the extraction of quantitative features from medical images followed by the conversion of radiological information into mineable high-dimensional data.^4,5^ Radiogenomics is an emerging area within radiomics aimed at creating a bridge between phenotype and genotype. It investigates the correlation between quantitative radiomics features and the corresponding gene expression profiles.^1^ In this review, we will investigate the role of VB in abdominal pathology, focusing on its pipeline development and potential benefits.

## The Virtual Biopsy pipeline

### Model development

**Figure 2.**
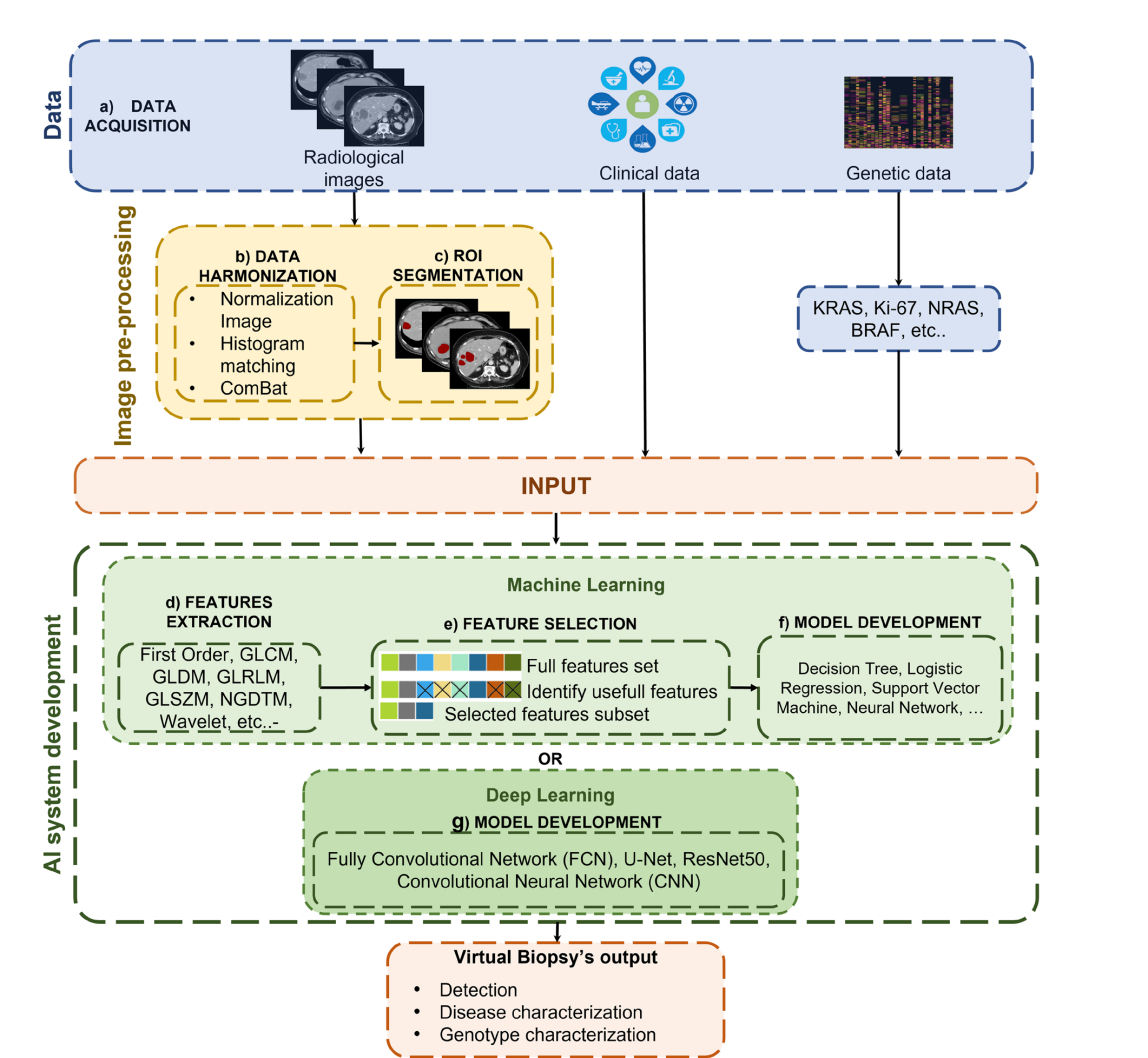
The VB steps are divided as following: (a) data collection, where radiological images, clinical and genetic data are acquired; (b) data hormonisation, where all data are normalised according different methods; (c) ROI segmentation: the radiological images are segmented according to the area of interest. The development of VB signature can be performed using Machine Learning, which includes the following steps: (d) features extraction, (e) features selection and (f) model development; if Deep Learning is used, the only necessary steps will be (g) model development. ROI, region of interest; VB, Virtual Biopsy.

The first step of the VB pipeline consists of the collection of data (Figure 2a). To develop a robust radiomics/radiogenomics model, multi-dimensional and multi-institutional data should be collected including high-quality annotated medical images, clinical, pathological, and molecular data. This will ensure generalisability across imaging protocols and patient populations. The second step consists of data harmonisation (Figure 2b) to reduce data variability that is performed either by normalisation, by histogram-matching—where intensity histograms are transformed to match a reference histogram—or, finally, by the ComBat method.^6^ To achieve a reliable output, the data set is divided into groups: the first group is used to train the model, the second to test and fine-tune it, and the last for its validation. Validation data sets can be internal, when applied in a similar clinical setting and population of the training set, or external, when applied in different clinical settings or populations with different characteristics, *e.g.* with varying disease prevalence.^4,7^ External validation ensures wider generalisability of the model.

Once the image data sets have been pre-processed, lesions are highlighted by tracing either 2D regions of interest (ROIs) or 3D volume of interest (VOIs) to differentiate them from the neighbouring structures (Figure 2c). This task is called “ROI Segmentation” and can be either manual, semi-automatic, or fully automatic. Manual and semi-automatic segmentation methods are time-consuming and may lead to high intra- and interobserver variability.^8^ Moreover, the development of automatic segmentation systems is hampered by the lack of large annotated data sets, large variability in cancer and organ shapes and tissue heterogeneity.

The third step in the workflow is features extraction (FE) from the segmented ROIs (Figure 2d). Typically in the initial stage, a large number of features is extracted including: (a) first-order features, from gray-level intensity histograms and lesion shape; b) second-order features, related to the spatial relationship between pixels, calculated using different matrices, *e.g.* gray-level co-occurrence (GLCM), gray-level run-length (GLRLM), gray-level dependence (GLDM), gray-level size zone (GLSZM), neighbouring gray tone difference (NGTDM); (c) transform-based features, *e.g.* Wavelet, Gabor, Laws, Laplacian.

A features selection (FS) step will allow (Figure 2e) to select, as input of the model, only features that are reproducible, non-redundant, and relevant for the task. Moreover, FS is crucial to develop robust and generalisable models, since the higher the number of features in a model and/or the lower the number of cases, the higher the risk of overadaptation of the model to the training data, in other words of overfitting.^4^ Finally, FS will reduce the computational cost, while improving the performance of the model.^9^ Some FS methods use a score, based on the relationship between each feature and the desired output, evaluated using statistical techniques (*e.g.* area under the receiver operating characteristic (ROC) curve (AUC), Mann–Whitney *U* test, Pearson χ^2^ test). Others are based on machine learning (ML), where FS is performed automatically by one of the following methods: genetic algorithms,^10^ maximum relevance minimum redundancy,^11,12^ affinity propagation,^10^ least absolute shrinkage, and selection operator.^13–16^ Finally, in the last step (Figure 2f), the radiomics methods are developed using algorithms such as logistic regression,^14,17^ k-nearest neighbour,^18^ naïve Bayes classifier,^19^ support vector machines,^20,21^ random forest,^22,23^ neural network^24,25^ and deep learning (DL).^26,27^ The main characteristic of DL is that it does not require the steps of FE and FS, since information is automatically extracted from the images by the algorithm (Figure 2g).

### Clinical validation

After developing and tuning the model on the training and testing data sets, the algorithm is validated on different data sets, to evaluate its robustness, reliability, and generalisability. The goal of the clinical validation is to assess if VB can detect and/or characterise tissues in a similar way to conventional biopsy, to drive treatment choice, and to predict patient outcomes. For the above reasons, a robust and reliable gold-standard should be always available, as the result of conventional biopsy or additional tests performed on the tissues.

Algorithm performances can be assessed using different metrics, *i.e.* accuracy, sensitivity, specificity, negative- and positive-predictive value. However, one of the most used metrics in clinical validation is the ROC curve analysis. This method consists in computing the ROC curve, a graphical plot that illustrates the true-positive rate against the false-positive rate at various threshold settings given by a classifier system and measuring the area under the curve (AUC), evaluating the probability that the model ranks a random positive example more highly than a random negative one. In cases where there are wide disparities between classification groups, AUC values may not be a reliable metric since it doesn’t differentiate false-negative from false-positive elements. AUC ranges between 0 and 1, where 1 indicates the 100% correct prediction.

In literature, most VB studies rely on retrospective data collection. In such conditions, algorithm performances might be overestimated since patients enrolled for both model development and validation fulfilled specific inclusion criteria, which may not be representative of a global clinical reality. Efforts should be made to overcome the issue to develop more generalisable models. One way to accomplish this task is to enrol patients prospectively. Indeed, in prospective cohort studies, although some participants might harbour risk factors, the group of interest does not have a confirmed clinical outcome. Only prospectively validated radiomics models will have the opportunity to be introduced into clinical practice.

## Methods and materials

This review provides an overview of VB applications in abdominal oncologic pathology. Relevant articles, published from January 2019 to December 2021, were identified by searching on Google Scholar and PubMed. Searches were manually supplemented, and retrieval of any additional articles meeting eligibility criteria was included in the reference list. Keywords used to select articles were: virtual biopsy, radiomics, abdominal organ (prostate, pancreas, liver, bladder, colorectal, uterus, gynaecological, kidney, gastrointestinal stromal tumour (GIST)), detection, characterisation, and radiogenomics. Published studies fulfilling the following criteria were included: (i) oncologic-related; (ii) the gold-standard was the biopsy outcome; (iii) the aim of the paper was at least one of the following: detection, characterisation, radiogenomic analysis of the disease; (iv) the VB system radiomics pipeline was described in detail, including the feature extraction method, selection, and development of ML and/or DL models; (v) the study was written in English; (vi) the developed model was validated (either internally or externally).

Using this search strategy, 63 out of 109 articles met the inclusion criteria and were considered relevant for this review. Among them, 10 were related to the prostate, 13 to the female pelvic area, 30 to the gastrointestinal (GI) tract, and 10 to miscellaneous organs.

No ethical approval was required for this study.

## Results

### Gastroenteric tract

#### Gastrointestinal stromal tumour

GISTs VB studies are presented in Supp Table 1.^11,28–33^ GISTs are the most common of the rare non-epithelial neoplasms of the GI tract, accounting for 0.1–3% of malignancies^34^ and are classified into four groups, very-low, low, intermediate, and high risk of cancer, according to pathological tumour size, location, and mitotic count. Treatment depends on risk category and disease stage. VB biomarkers have been developed to classify patient risk, taking into account lesion heterogeneity. In particular, Song et al^11^ and Zhang et al^28^ developed models based on CT scans, capable of stratifying patients between low and high risk, yielding an AUC of 0.85 and 0.94, respectively. Other studies, such as those from Zhang et al^30^ and Zhao et al^29^ developed radiogenomics models to evaluate Ki-67 expression, to predict disease-specific survival and risk of recurrence. Of the above, preliminary assessment of the Ki-67 expression was the most promising (AUC = 0.78 in both studies).

#### Colorectal cancer

VB studies on colorectal cancer (CRC) are presented in Supp Table 2.^35–43^ In the context of CRC, VB could provide information on tumour stage and grade. To this point, Ma et al^40^ developed an MRI-based radiomics model dichotomising CRC patients into poor and high-to-moderate histological grade, and into T1-2 and T3-4 stages, resulting in an AUC of 0.86 and 0.81, respectively.

Regarding genotype characterisation, efforts have been made toward the detection of carriers of BRAF and RAS (KRAS and NRAS) gene mutations, which are usually associated with shorter disease-free and overall survival, that however may benefit from tailored therapies. Assessment is usually performed through genetic molecular profiling of tissue biopsy which carries several drawbacks, as discussed in previous sections. Cui et al^35^ were able to demonstrate that radiomics can predict RAS and BRAF mutation status in patients with CRC with acceptable performances (AUC of 0.74).

### Liver cancer

VB studies on primary neoplasms, the most common being hepatocellular carcinoma (HCC), and liver metastases are reported in Supp Table 3.^18,44–49^ Wu et al^46^ developed a VB model to classify patients with HCC according to tumour stage by integrating radiomics and clinical features. The model they developed showed encouraging results (AUC = 0.80). VB approaches were also proposed to explore the relationships between the immune cell microenvironment and tumour initiation, progression, and dissemination. For example, Hectors et al^49^ assessed early HCC recurrence predicting values of radiomics and genomics features to immunotherapy targets (CTLA-4 and PD-1), yielding promising results (AUC = 0.76). There is a need to develop improved methods to accurately predict the gene-mutational status of liver metastases, to select the best treatment for each patient, paving the way to precision oncology. One example was provided by Shi et al,^18^ who investigated whether radiomics and/or semantic features could classify patients with CRC liver metastasis according to their mutational status. They found that, based on the status of RAS and BRAF, they were able to develop and validate a combined score to distinguish between mutant and wild-type lesions, yielding promising performances (AUC = 0.79).

### Pancreatic cancer

VB studies on pancreatic cancer are reported in Supp Table 4.^22,23,50–57^ Pancreatic adenocarcinoma (PDAC) comprises 90% of pancreatic neoplasms, the remaining being pancreatic neuroendocrine tumours (PNET; 2–10%) and other rare subtypes. Due to the late onset of symptoms, most patients with PDAC are diagnosed with locally advanced (30–35%) or metastatic (50–55%) disease,^58^ while smaller PDACs (≤2 cm) are inconspicuous and may evade detection. VB could potentially allow the detection of pancreatic cancer in an earlier stage when the disease is still curable. Several AI models have been proposed for this purpose. For example, Chen et al^51^ developed a CT-based model to investigate whether ML radiomics could differentiate between PDAC and non-cancerous tissue with encouraging performances (accuracy of 0.86). Once the cancer diagnosis has been made, it will be necessary to determine the tumour grade. Preliminary studies have shown promising results in the classification both of PDAC and PNET with VB. To this point, Gu et al^23^ developed and validated a nomogram, which includes both radiomics and clinical features, to non-invasively classify Grade 1 *vs* 2/3 PNET patients.

Intraductal papillary mucinous neoplasms (IPMNs) represent a heterogeneous group of cystic pancreatic neoplasms. The management of IPMN remains controversial. Until not long ago, surgical resection was recommended to prevent the onset of malignant pancreatic cancer. Unfortunately, this strategy has led to overtreatment and postoperative complications, leading to an increase in the risk of co-morbidity and mortality of 50 and 4% of cases, respectively.^50^ Current guidelines recommend the maintenance of a balance between the risk of potential malignant transformation and the risks of pancreatic resection,^59^ suggesting that only patients with high-grade dysplasia (HGD) should undergo surgery.^60^ In this context, the VB system to pre-operatively assess IPMN grade could support the decision process, reducing the drawbacks of an invasive biopsy. An example is given by Tobaly et al,^50^ who developed a CT-based model to differentiate between LGD, HGD, and invasive IPMN. Despite the challenge of constructively integrating clinical–biological and radiological data, their radiomics model showed encouraging performances (AUC = 0.71) in a large validation set (*n* = 112) and was able to reliably differentiate the different IPMN grades, particularly benign (low-grade dysplasia) from malignant (high-grade dysplasia and invasive carcinoma) ones, potentially contributing to better patient management.

### Urogenital tract

#### Kidney cancer

Renal cell carcinoma (RCC) comprises three main histopathological subtypes: clear cell (ccRCC) (90% incidence; poorer prognosis), papillary (pRCC), and chromophobe (cRCC), the former two representing 80–90% of all cases.^61,62^ Diagnosis of different subtypes is not readily obtained by imaging and biopsy, which plays a key role in confirming cancer diagnosis, carries a 14% non-diagnostic rate in patients with small renal masses and has a low negative predictive value (70%) in ruling-out cancer. VB has been proposed to overcome some of the limitations of the current diagnostic pipeline (Supp Table 5).^12,62–65^ Indeed, Said et al^62^ successfully implemented an ML-based model to differentiate RCC from benign tissue and to classify renal masses into different histotypes based on MRI examinations.

The current standard of care for the management of RCC is partial, or radical nephrectomy. However, the rising incidence and increasing detection of small lower risk RCC have led to alternative less invasive treatment options, such as percutaneous ablation or lesion monitoring within wait-and-see or active surveillance programmes. In this context, pre-treatment assessment of tumour aggressiveness is of key importance for clinical decision-making. Based on MRI, Purkayastha et al^12^ developed a non-invasive VB model to differentiate between low- and high-grade RCC, yielding an AUC of 0.59. Also, Gurbani et al^65^ were able to discriminate kidney cancer grade on CT scans, with an AUC of 0.67 in the internal validation set. Further analysis should be carried out on multicentric data sets and by combining clinical and/or pathological features in the ML model.

#### Bladder cancer

Bladder cancer (BCa) can be stratified into low- and high-grade based on the presence or not of invasion of the muscle layer. Unfortunately, grade assessment is not always conclusive. Moreover, although most non-muscle-invasive bladder cancers (NMIBCas) are low grade and have an indolent natural history, approximately 20–25% of NMIBCas may progress locally, with invasion of the muscolaris propria, or develop distant metastases.^66–69^ Currently, cystoscopy together with histological evaluation of the resected tissues is the mainstay of diagnosis and clinical staging of BCa. However, as biopsy is unlikely to sample every part of the tumour, staging may be inaccurate, and up to 25% of muscle-invasive bladder cancers can be initially misdiagnosed as NMIBCas. Repeated examinations improve the diagnostic yield, but this implies higher costs and may be distressing for the patient.

Supp Table 6^66–70^ reports on several studies addressing the assessment of involvement of the bladder wall with VB. Of note, Zhang et al^67^ developed a non-invasive DL-based VB model to pre-operatively stratify patients according to the muscle-invasive status of BCa. Their model, applied on the external validation set, outperformed the radiologists in terms of accuracy (reader1 = 0.74. reader2 = 0.57, DL model = 0.75) and specificity, while sensitivity was lower. It could well be that radiologists in the study had a higher propensity to report the invasion of the muscular layer due to their fear of the consequences of missing MIBC. Regarding tumour grade staging, Wang et al^66^ proposed a radiomics model to pre-operatively discriminate low- and high-grade BCa tumours, reaching high performances (AUC = 0.93). Finally, concerning BCa genotype characterisation, Lin et al^70^ developed a nomogram incorporating radiomics, clinicopathological parameters, and RNA-sequence data for predicting the overall survival of patients with bladder urothelial carcinoma (BLCA), yielding an AUC of 0.96.

#### Ovarian cancer

VB studies on ovarian cancer (OC) are reported in Supp Table 7.^16,17,71–77^ Epithelial OC (EOC) is the most common and lethal among gynaecologic malignancies since more than 80% of cases are diagnosed at an advanced stage.^78^ Currently, diagnosis and subtype classification of EOC into the serous, mucinous, clear-cell, and endometrioid variants is obtained by incisional or aspiration biopsy. Non-invasive assessment with VB could provide relevant information to the clinicians, potentially useful for treatment planning. A VB application example is provided by Pan et al,^17^ which have developed a classification system based on radiomics and semantic features to distinguish serous and mucinous pathological types in patients with pathology-confirmed ovarian cystadenoma, yielding excellent results (AUC of 0.92).

EOC is classified into two categories having different clinicopathological and molecular features. Type I is characterised by indolent behaviour and when confined to the ovary has an excellent prognosis, while Type II has a far more aggressive behaviour, resulting in a poor overall prognosis. To non-invasively differentiate between these two categories, an MRI-based radiomics model has been developed and externally validated (AUC = 0.85) by Jian et al.^71^ The authors were able to identify the most critical regions for differentiating between Type Ι and Type ΙΙ EOC, *i.e.* the border zone between the solid and cystic components and the less compact area of the solid component.

Few studies have integrated genetic features into radiomics signatures during model development. These studies explore the correlation between biological information, molecular signalling pathways, and tumour microenvironment, integrating radiomics features and genetic data. Meier et al,^77^ *e.g.* searched for the presence of associations between morphology-related radiomics features (GLCM) and BRCA mutational status (BRCA1, BRCA2 and negative) in high-grade serous ovarian carcinoma (HGSOC) patients that underwent CT. Unfortunately, they did not find significant associations. Further analysis of other radiomics feature subgroups and advanced computational analysis will be required to quantitatively analyse phenotypic traits on standard of care CT.

#### Cervical and endometrial cancer

Among gynaecologic malignancies, endometrial cancer (EC) is characterised by a good prognosis, having a 5-year patient survival rate of 84%. However, if incorrectly staged, low-risk patients may undergo unnecessary surgery with overtreatment.^79^ Opposite, high-risk patients could be undertreated, with a dismal survival rate. EC patients could therefore benefit from precision oncology diagnostic tools. Supp Table 8^15,25,80^ presents studies on VB models for assessing tumour aggressiveness and evaluating genetic correlations in patients with EC. In particular, Fasmer et al^80^ developed an MRI-based model to non-invasively assess EC aggressiveness. Their radiomic signatures yielded promising results, proving the innovative ability of the VB to capture relevant markers from the whole volume, compared to the traditional biopsy which provides the outcome of a specimen. Moreover, Veeraraghavan et al^15^ developed a radiogenomics VB tool to non-invasively identify DNA mismatch repair deficient (MMR-D) and tumour mutational burden-high (TMB-H) in EC patients from CT. They were relatively accurate in identifying both MMR-D (AUC = 0.78) and TMB-H (AUC = 0.87). In conclusion, VB may provide an adjunct tool to molecular profiling, given its potential advantage in the setting of intratumor heterogeneity.

#### Prostate cancer

Prostate cancer (PCa) diagnosis is now greatly supported by multiparametric MRI.^81^ Unfortunately, PCa detection is affected by the radiologist’s experience, scanning protocol, and MR equipment.^13^ In this context, VB could undoubtedly bring a benefit to the patient. The opportunity to differentiate benign hypertrophy, inflammation, or normal prostatic tissue from PCa could allow the biopsy needle to be directed toward the most suspicious areas within the gland. Automatic lesion and prostate segmentation could make it easier and faster to perform fusion biopsy or could allow accurate radiotherapy planning, boosting the dose to cancer lesions. Moreover, biopsy is well known to underestimate, or overestimate disease grade due to lesion heterogeneity. Also, visual MRI assessment is not supportive, being not sufficiently granular to detect different levels of lesion aggressiveness.

Recent publications on prostate VB applications, presented in Supp Table 9,^10,13,14,24,82–85^ have shown that MRI-based VB models can distinguish cancer from benign prostatic tissue with an accuracy, evaluated by ROC analysis, between 0.89 and 0.94.^14^ Further testing will have to be performed in a clinical environment to assess the performances of radiologists by adding the information provided by VB.^86^ Large data collections of high-quality and well-annotated MRI examinations and metadata are being implemented, allowing a significant breakthrough.^87^

Just as tissue biopsy, VB may guide treatment selection in the future. Patients with indolent cancer, if correctly diagnosed, could be spared whole gland treatments. To this point, Giannini et al^13^ developed a fully automated computer-aided diagnosis system to localise, segment, and stratify PCa according to its aggressiveness. This prototype, which was externally validated on data from different MRI scanners yielded encouraging results (AUC = 0.81). Similarly, Nicoletti et al^10^ developed a radiomic model to distinguish between aggressive (GG ≥ 3) and indolent (GG ≤ 2) PCa, based on bi-parametric MRI, yielding similar results. Lastly, Woźnicki et al^82^ developed an ML-based model to categorise between: (a) histologically proven PCa and benign prostate lesions; (b) clinically significant (GG ≥ 2) *vs* non-significant PCa. The main finding of the latter study was that the ML model showed higher predictive performance in comparison to the PIRADS as well as clinical biomarkers, such as PSA density and digital rectal examination.

## Conclusions

This review discusses the evolving role of VB as an alternative to traditional tissue biopsy. VB has shown promise in abdominal pathology in particular to detect and grade tumours, and may also provide information on their mutational status. Advantages of VB include its low cost, the opportunity to assess the entire lesion, providing information on its heterogeneity, its non-invasiveness, and its short turnaround time. However, VB has the disadvantage of having a low spatial and contrast resolution, with respect to tissue biopsy that is able to explore processes at a subcellular level. For the above reasons, VB will never substitute tissue biopsy but will more probably limit its use to well-selected patients where imaging does not provide a conclusive diagnosis. The development of VB biomarkers is held back by the lack of large databases providing well-annotated and high-quality images, and their accompanying metadata, including robust reference standards, which limit the opportunity of performing extensive validation studies. Indeed, the variability introduced using different scanners, software, and acquisition protocols, as well as the different examined populations, may limit the generalisability and reproducibility of the results.

In the future, more reliable holistic biomarkers will be developed by integrating the information derived from VB with that of other omics, paving the way to a personalised, precision approach to each patient.

## Supplementary Material

SupplementaryMaterials
