# Supplementary material for: Virtual biopsy in abdominal pathology: where do we stand?
Source: BJR Open. 2023 Feb 28;5(1):20220055. doi: 10.1259/bjro.20220055 (PMC10077420; doi:10.1259/bjro.20220055)
Supplement: SupplementaryMaterials [file SupplementaryMaterials.docx]

S-TABLE I: PAPERS RELATED TO THE GIST

| Paper | First Author | Aims | Dataset | Features Extracted | Feature Selection | Classifier | Results  (best performing model) |
| --- | --- | --- | --- | --- | --- | --- | --- |
| (11) | Song Y. | To predict the malignancy degree of GIST | 500 CT  (2 centres) | n=396 (First Order, GLCM, GLRLM, Form Factor, Haralic) | MRMR, LASSO (n=13) | SVM | AUC=0.85, acc=0.81 (external validation) |
| (28) | Zhang Q. | To predict the malignancy degree of GIST | 370 CT  (4 centres) | n=833 (First Order, GLCM, GLRLM, GLSZM, GLDM, NGTDM) | MRMR, LASSO (n=43) | LR | AUC=0.94, acc=0.89 (external validation) |
| (29) | Zhao Y. | To predict Ki-67 expression in GIST cancer | 344 CT  (3 centres) | n=704 (First Order, GLCM, GLRLM, GLSZM, GLDM, NGTDM) | Boruta algorithm (n=21) | RF | AUC=0.78 (external validation) |
| (30) | Zhang Q. | To predict Ki-67 expression in GIST cancer | 339 CT  (4 centres) | n=833 (First Order, GLCM, GLRLM, GLSZM, GLDM, Wavelet) | ICC & MRMR & LASSO (n=6) | LR | AUC=0.78 (external validation) |
| (31) | [Wang M.](https://pubmed-ncbi-nlm-nih-gov.bibliopass.unito.it/?term=Wang+M&cauthor_id=33968714) | For risk stratification of GIST | 324 CT  (3 centres) | n=704 (First Order, GLCM, GLRLM, GLDM) | Spearman correlation, Boruta algorithm (n=10) | LR, SVM, RF | AUC=0.90 (external validation) |
| (32) | Chu H. | For risk stratification of GIST cancer | 292 CT (monocentric) | n=396 (First Order, GLCM, GLRLM, GLSZM) | Spearman method, LASSO (n=2) | LR | AUC=0.79, sens=0.84, spec=0.69, acc=0.76 (internal validation) |
| (33) | Zhang L. | (a)To discriminate between advanced and non advanced GIST cancer  (b)For the risk stratification | 140 CT (monocentric) | n=396 (First Order, GLCM, GLRLM, GLSZM) | Pearson correlation, RF (n=5) | RF | (a) AUC=0.94, acc=0.90. (b) AUC=0.81, acc=0.67 (internal validation) |

Note: gastro-intestinal stromal tumour (GIST), Computer Tomography (CT), Magnetic Resonance Imaging (MRI), gray-level co-occurrence (GLCM), gray-level run-length (GLRLM), gray-level dependence (GLDM), gray-level size zone (GLSZM), neighbouring gray tone difference (NGTDM), maximum relevance minimum redundancy (MRMR), Wilcoxson (WLCX), Intra-Class Correlation (ICC), least absolute shrinkage and selection operator (LASSO), Logistic Regression (LR), Support Vector Machines (SVM), k-nearest neighbour (KNN), Random Forest (RF), Decision Tree (DT), Neural Network (NN), Area Under the Curve (AUC), positive predictive value (PPV), negative predictive value (NPV), sensibility (sens), specificity (spec), accuracy (acc), Not Available (N.A.).

S-TABLE II: PAPERS RELATED TO THE RECTAL CANCER

| Paper | First Author | Aims | Dataset | Features Extracted | Feature Selection | Classifier | Results  (best performing model) |
| --- | --- | --- | --- | --- | --- | --- | --- |
| (35) | Cui Y. | To predict KRAS mutation status in patients with rectal cancer | 390 MRI (2 centres) | n=960 (First Order, GLCM, GLRLM, GLDM, GLSZM, Wavelet) | univariate statistical test (n=7) | LR, DT; SVM | AUC=0.74 (external validation) |
| (36) | Meng X. | To evaluate biological characteristics of rectal cancer: a) lymph node metastasis, b) tumour differentiation, c) fraction of Ki-67 positive tumour cells, d) human epidermal growth factor receptor 2 (HER-2), and e) KRAS-2 gene mutation status. | 345 MRI (monocentric) | n= 644( First Order, GLCM, time-intensity, Wavelet and clinical features) | MRMR and WLCX, RF, SVM and LASSO  (n=N.A.) | LR | a) AUC= 0.67 ; b) AUC=0.72; c) AUC=0.70; d) AUC= 0.70; e) AUC=0.65  (internal validation) |
| (37) | Shu Z. | Detection rectal cancer liver metastases, based on primary rectal cancer | 194 (MRI)  (monocentric) | n=328 (First Order, GLCM, GLRLM, Form Factor) | Spearman & LASSO (n=7) | LR | AUC=0.83 (internal validation) |
| (38) | Liu H. | To predict likelihood of developing distant metastases | 177 MRI (monocentric) | n=385 (First Order, GLCM, GLRLM and clinical features) | ICC, univariate statistical test, RF (n=10) | LR | AUC=0.83, sens=0.72, sepc=0.94, acc=0.87 (internal validation) |
| (39) | Lu HC. | To differentiate between high and low stage rectal tumours | 174 MRI (monocentric) | n=12 (First Order, GLCM, GLRLM, Wavelet) | / | LR | AUC=0.79 (internal validation) |
| (40) | Ma X. | a) To predict the degree of differentiation of the CRC b) To predict T stage | 152 MRI (monocentric) | n=1029 (First Order, GLCM, GLSZM, GLRLM, NGTDM, Wavelet) | Least Absolute shrinkage & LASSO (n=15) | multilayer perceptron (MLP), LR, SVM, DT, RF, K-NN | a) AUC=0.86, sens=0.83, spec=0.85;  b) AUC=0.81, sens=0.76, spec=0.74 ;  (internal validation) |
| (41) | Negreros-Osuna AA. | To detect BRAF mutant colorectal tumours | 145 CT (monocentric) | n=6 (First Order) | N.A. | recox regression | The Standard Deviations and Mean value Positive Pixels of radiomics texture features were significantly lower. |
| (42) | Sun Y. | To differentiate between high and low stage rectal tumours | 119 MRI (monocentric) | n=256 (First Order, GLCM, GLRLM, Wavelet) | LASSO (n=N.A.) | nomogram | AUC=0.85, sens=0.79, spec=0.82 (internal validation) |
| (43) | Oh JE. | To predict KRAS mutation status in patients with rectal cancer | 60 MRI (monocentric) | n=24 (First Order, GLCM, GLRLM, Laplacian ) | t-test (n=3) | DT | sens=0.84, spec=0.80, acc=0.82 (internal validation) |

Note: gastro-intestinal stromal tumour (GIST), Computer Tomography (CT), Magnetic Resonance Imaging (MRI), gray-level co-occurrence (GLCM), gray-level run-length (GLRLM), gray-level dependence (GLDM), gray-level size zone (GLSZM), neighbouring gray tone difference (NGTDM), maximum relevance minimum redundancy (MRMR), Wilcoxson (WLCX), Intra-Class Correlation (ICC), least absolute shrinkage and selection operator (LASSO), Logistic Regression (LR), Support Vector Machines (SVM), k-nearest neighbour (KNN), Random Forest (RF), Decision Tree (DT), Neural Network (NN), Area Under the Curve (AUC), positive predictive value (PPV), negative predictive value (NPV), sensibility (sens), specificity (spec), accuracy (acc), Not Available (N.A.).

S-TABLE III: PAPERS RELATED TO LIVER CANCER

| Paper | First Author | Aims | Dataset | Features Extracted | Feature Selection | Classifier | Results  (best performing model) |
| --- | --- | --- | --- | --- | --- | --- | --- |
| (44) | Xu X. | To predict MVI and clinical  outcomes in patients with HCC. | 495 CT  (monocentric) | n=1210 (First Order, GLCM, GLRLM, GLSZM, GLDM, NGTDM, wavelet+clinical variables) | ICC, Recursive SVM (n=8) | LR | AUC=0.84, sens=0.90, spec=0.79, acc=0.83 (internal validation) |
| (45) | Jian H. | To assess the diagnostic accuracy of a radiomics models for HCC in high-risk patients | 211 MRI  (monocentric) | n=N.A (First Order, GLCM, GLRLM, Haralick correlation) | LASSO (n=N.A) | LR | AUC=0.81, sens=0.73, spec=0.77, PPV=0.91, NPV=0.47 (internal validation) |
| (46) | Wu M. | To discriminate high-grade and low-grade HCC | 170 MRI  (monocentric) | n=328 (First Order, GLCM, GLRLM, GLSZM, GLDM, NGTDM, wavelet+clinical features) | LASSO (n=20) | LR | AUC=0.80 (internal validation) |
| (47) | Ma X. | To predict MVI in patients with HCC | 157 CT  (monocentric) | n=647 (First Order, GLCM, GLRLM, GLSZM, NGTDM+clinical features) | LASSO (n=21) | SVM | AUC=0.82 (internal validation) |
| (48) | Zhu Y. | To predict MVI in patients with HCC | 142 MRI  (monocentric) | n=58 (First Order, GLCM, GLRLM*+*clinical variables) | t-test, LR, Pearson correlation (n=9) | LR | AUC=0.81, sens=0.81, spec=0.79 (internal validation) |
| (49) | Hectors S. J. | To early predict immuno-oncologic characteristics of HCC | 48 MRI (monocentric) | n=218 (First Order, GLCM, GLRLM, GLSZM, GLDM, Wavelet + clinical/genetic variables) | N.A. | LR | AUC=0.76 (internal validation) |
| (18) | Shi R. | Prediction of KRAS, NRAS and  BRAF status in colorectal cancer patients with liver metastasis using a deep artificial neural network based on radiomics and semantic features | 159 CT  (monocentric) | n=851 (First Order, GLCM, GLRLM, GLSZM, GLDM, NGTDM, clinical variables) | ICC (n=N.A) | NN | AUC=0.79, sens=0.70, spec=0.75, PPV=0.84, NPV=0.56 (internal validation) |

Note: hepatocellular carcinoma (HCC), microvascular invasion (MVI), Computer Tomography (CT), Magnetic Resonance Imaging (MRI), gray-level co-occurrence (GLCM), gray-level run-length (GLRLM), gray-level dependence (GLDM), gray-level size zone (GLSZM), neighbouring gray tone difference (NGTDM), maximum relevance minimum redundancy (MRMR), Wilcoxson (WLCX), Intra-Class Correlation (ICC), least absolute shrinkage and selection operator (LASSO), Logistic Regression (LR), Support Vector Machines (SVM), k-nearest neighbour (KNN), Random Forest (RF), Decision Tree (DT), Neural Network (NN), Area Under the Curve (AUC), positive predictive value (PPV), negative predictive value (NPV), sensibility (sens), specificity (spec), accuracy (acc), Not Available (N.A.).

S-TABLE IV: PAPERS RELATED TO PANCREATIC CANCER

| Paper | First Author | Aims | Dataset | Features Extracted | Feature Selection | Classifier | Results  (best performing model) |
| --- | --- | --- | --- | --- | --- | --- | --- |
| (50) | Tobaly D. | For differentiating  benign from malignant intraductal papillary mucinous neoplasms of the pancreas | 408 CT  (51 centres) | n=107 (First Order, GLCM, GLSZM, GLRLM) | Pearson correlation & LASSO (n=11) | LR | AUC=0.71, sens=0.69, spec=0.57  (external validation) |
| (51) | Chen PT. | Detection pancreatic cancer | 1379 CT  (3 centres) | n=88 (First Order, GLCM, GLDM, GLSZM, GLRLM, NGTDM) | XGboost (n=14) | XGboost | sens=0.86, spec=1.00, acc=0.86, AUC=0.91  (external validation) |
| (23) | Gu D. | To assess pancreatic cancer grade | 138 CT  (2 centres) | n=853 (First Order, GLCM, GLRLM, GLSZM, GLDM, NGTDM, Wavelet) | MRMR (n=25) | RF | AUC= 0.90 (external validation) |
| (52) | Wei R. | To differentiate pancreatic cystic neoplasms | 260 CT (monocentric) | n=409 (First Order, GLCM, GLRLM, GLSZM, NGTDM, GLDM, wavelet + clinical and morphological features) | LASSO (n=22) | SVM | AUC=0.84, sens=0.67, spec=0.82 (internal validation) |
| (53) | Chu LC. | Detection pancreatic cancer | 191 CT (monocentric) | n=478 (First Order, GLCM, GLSZM, GLRLM, GLDM, NGTDM, Wavelet) | MRMR (n=5) | RF | sens=0.95, spec=0.92, acc=0.97  (internal validation) |
| (22) | Park S. | To distinguish autoimmune pancreatitis from pancreatic ductal adenocarcinoma | 182 CT (monocentric) | n=431(First Order, GLCM, GLRLM, Wavelet) | MRMR (n=35) | RF | TP=0.95 (thin-slice venous phase), TP=0.84 (thin-slice arterial phase), TP=0.77 (thick-slice venous phase) (internal validation) |
| (54) | Bian Y. | To assess pancreatic cancer grade | 139 MRI (monocentric) | n=2126 (First Order, GLCM, GLRLM, GLSZM, GLDM, NGTDM, Wavelet) | Variance analysis, Wilcoxon rank-sum test, MRMR, LASSO (n=14) | LR | AUC= 0.73 (internal validation) |
| (55) | Ren S. | Detection mass-forming pancreatitis from pancreatic ductal adenocarcinoma | 109 CT  (monocentric) | n=396 (First Order, GLCM, GLSZM, GLRLM) | MannWhitney test & MRMR & RF (n=10) | RF | sens=0.83, spec=0.81, acc=0.82 (internal validation) |
| (56) | Zhao Z. | To assess pancreatic cancer grade | 99 CT (monocentric) | n=585 (First Order and Wavelet) | MRMR (n=24) | SVM | AUC= 0.88, sens=0.84, spec=0.89 (internal validation) |
| (57) | Attiyeh M. A. | To predict overall survival and genetic mutations of pancreatic cancer | 35 CT (monocentric) | n=255 (First Order, GLCM, GLRLM, Wavelet, local binary patterns, and angle co-occurrence matrices) | fuzzy MRMR (n=32) | LR | The number of genes altered was the only significant predictor (p = 0.016). The prediction model achieved an R^2=0.73 and mean prediction error of 10%. Radiomics may predict genetic mutations. (internal validation) |

Note: Computer Tomography (CT), Magnetic Resonance Imaging (MRI), gray-level co-occurrence (GLCM), gray-level run-length (GLRLM), gray-level dependence (GLDM), gray-level size zone (GLSZM), neighbouring gray tone difference (NGTDM), maximum relevance minimum redundancy (MRMR), Wilcoxson (WLCX), Intra-Class Correlation (ICC), least absolute shrinkage and selection operator (LASSO), Logistic Regression (LR), Support Vector Machines (SVM), k-nearest neighbour (KNN), Random Forest (RF), Decision Tree (DT), Neural Network (NN), Area Under the Curve (AUC), positive predictive value (PPV), negative predictive value (NPV), sensibility (sens), specificity (spec).

S-TABLE V: PAPERS RELATED TO THE KIDNEY CANCER

| Paper | First Author | Aims | Dataset | Features Extracted | Feature Selection | Classifier | Results  (best performing model) |
| --- | --- | --- | --- | --- | --- | --- | --- |
| (12) | Purkayastha S. | To predict ccRCC grading in the kidney | 439 MRI  (5 centres) | n=3087 (First Order, GLCM, GLRLM, GLSZM, NGTDM, GLDM) | Chi-square Test, MRMR (n=3) | LR, NN, DT, Boosting, Bayesian, Bagging, RF, SVM, Linear Discriminant Analysis, k-NN | AUC=0.59, acc=0.77, sens=0.38, spec=0.86 (external validation) |
| (63) | Zeng H. | a) To assess molecular mutations: I) VHL, II) BAP1, III) PBRM1, IV) SETD2;  b) To predict molecular subtypes: I) m1, II) m2, III) m3, IV) m4.  c) 5-year survival in clear cell renal cell carcinoma | 382 CT  (multicentric public databases) | n=107 (First Order, GLCM, GLDM, GLRLM, GLSZM, NGTDM + proteomics, genomics, and transcriptomics features) | LASSO, RF, XGBoost  (n=N.A.) | RF, AdaBoost, LR, DT, SVM, NB, KNN | a.I) AUC=0.97, a.II) AUC=0.95, a.III) AUC=0.97, a.IV) AUC=0.95;  b.I) AUC=0.97, b.II) AUC=0.97, b.III) AUC=0.96, b.IV) AUC=0.95; c) AUC=0.85  (internal validation) |
| (64) | Bai X. | To predict synchronous distant metastases in patients with ccRCC | 201 MRI  (4 centres) | n=1029 (First Order, GLCM, GLDM, GLRLM, GLSZM, NGTDM, Wavelet+clinical features) | t-test, correlation variance, LASSO  (n=9) | nomogram | AUC=0.82  (external validation) |
| (65) | Gurbani S. | To assess kidney cancer aggressiveness | 141 CT (monocentric) | n=300 (First Order, GLCM) | Ranking  (n=6) | XGBoost, RF, SVM | AUC=0.67  (internal validation) |
| (62) | Said D. | a) To distinguish between Renal Cell Carcinomas (RCC) and benign tissue  b) To distinguish ccRCC and other lesions  c) To distinguish pRCC and other lesions | 125 MRI  (monocentric) | n=64 (First Order, GLCM) | MannWhitney  ( a) n=5, b) n=31, c) n=32) | RF | a) AUC=0.73, b) AUC=0.77, c) AUC=0.74  (internal validation) |

Note: clear cell renal cell carcinoma (ccRCC), papillary renal cell carcinoma (pRCC), Computer Tomography (CT), Magnetic Resonance Imaging (MRI), gray-level co-occurrence (GLCM), gray-level run-length (GLRLM), gray-level dependence (GLDM), gray-level size zone (GLSZM), neighbouring gray tone difference (NGTDM), genetic algorithms (GA), maximum relevance minimum redundancy (MRMR), affinity propagation (AP), Intra-Class Correlation (ICC), least absolute shrinkage and selection operator (LASSO), Logistic Regression (LR), Support Vector Machines (SVM), Random Forest (RF), Decision Tree (DT), naïve Bayesian (NB), Neural Network (NN), Deep Learning (DL), Area Under the Curve (AUC), sensibility (sens), specificity (spec), accuracy (acc), Positive Predictive Value (PPV), Negative Predictive Value (NPV), Not Available (N.A.).

S-TABLE VI: PAPERS RELATED TO BLADDER CANCER

| Paper | First Author | Aims | Dataset | Features Extracted | Feature Selection | Classifier | Results  (best performing model) |
| --- | --- | --- | --- | --- | --- | --- | --- |
| (67) | Zhang G. | To assess the bladder cancer muscle-invasiveness | 441 CT  (2 centres) | N. A | N. A. | Filter-guided Pyramid Network | AUC=0.79, sens=0.71, spes=0.77 (external validation) |
| (68) | Xu S. | To categorize between muscle-invasive bladder cancer and non−muscle-invasive | 218 MRI (monocentric) | n=156 (First Order, GLCM, GLRLM, GLSZM, NGTDM) | ICC & Boruta  (n=21) | RF | acc=0.90, F2 score=0.95 (internal validation) |
| (69) | Zheng J. | To categorize between muscle-invasive bladder cancer and non−muscle-invasive | 130 MRI (monocentric) | n=2602(First Order, GLCM, GLRLM, GLSZM, NGTDM, GLDM, Wavelet) | LASSO  (n=23) | LR | AUC=0.88 (internal validation) |
| (66) | Wang H. | To assess bladder cancer pathological grade | 70 MRI (monocentric) | n=924 (First Order, GLCM, GLRLM, GLSZM, NGTDM, GLDM) | t-test, LASSO  (n=7) | LR | AUC=0.93 (internal validation) |
| (70) | Lin P. | To predict the clinical outcome of bladder urothelial carcinoma | 62 CT  (monocentric) | n=1076 (First Order, GLCM, GLRLM, GLSZM, GLDM, Wavelet + n=1014 genes) | univariate LASSO Cox proportional hazard regression  (n=28) | Cox regression | The AUC = 0.96, sens=0.93, spec=0.90 (internal validation) |

Note: clear cell renal cell carcinoma (ccRCC), papillary renal cell carcinoma (pRCC), Computer Tomography (CT), Magnetic Resonance Imaging (MRI), gray-level co-occurrence (GLCM), gray-level run-length (GLRLM), gray-level dependence (GLDM), gray-level size zone (GLSZM), neighbouring gray tone difference (NGTDM), genetic algorithms (GA), maximum relevance minimum redundancy (MRMR), affinity propagation (AP), Intra-Class Correlation (ICC), least absolute shrinkage and selection operator (LASSO), Logistic Regression (LR), Support Vector Machines (SVM), Random Forest (RF), Decision Tree (DT), naïve Bayesian (NB), Neural Network (NN), Deep Learning (DL), Area Under the Curve (AUC), sensibility (sens), specificity (spec), accuracy (acc), Positive Predictive Value (PPV), Negative Predictive Value (NPV), Not Available (N.A.).

S-TABLE VII: PAPERS RELATED TO OVARIAN CANCER

| Paper | First Author | Aims | Dataset | Features Extracted | Feature Selection | Classifier | Results  (best performing model) |
| --- | --- | --- | --- | --- | --- | --- | --- |
| (71) | Jian J. | To differentiate between type I and type II epithelial ovarian cancer | 294 MRI  (8 centres) | n=851(First Order, GLCM, GLRLM, GLSZM, NGDM, GLDM, Wavelet) | Pearson chi-squared test & MRMR (n=4) | LASSO regression | AUC=0.85 (external validation) |
| (17) | Pan S. | To classify serous and mucinous pathological types in patients with ovarian cystadenoma | 103 CT  (2 centres) | n=396 (First Order, GLCM, GLRLM, GLZSM) | LASSO (n=5) | LR | AUC=0.92 (external validation) |
| (72) | Veeraraghavan H. | To predict outcomes in patients with high serous ovarian carcinoma | 75 CT  (multicentric public dataset) | n=75 (First Order, GLCM, GLRLM, GLSZM, GLDM, NGTDM + clinical/genetic variables) | N.A. | SVM | AUC=0.78 (external validation) |
| (16) | Zhang H. | (a)To categorize ovarian masses  (b)To categorize type I vs type II subtypes | 286 MRI (monocentric) | n=1714 (First Order, GLCM, GLRLM, GLSZM, NGTDM, GLDM, Wavelet) | LASSO ((a) n=84; (b) n=56) | LR | (a) AUC=0.95, acc=0.87, (b) AUC=0.85, acc=0.83 (internal validation) |
| (73) | Qi L. | a) Discriminate between benign and malignant and borderline cancers  b) Discriminate between malignant and borderline cancers | 265 US (monocentric) | n=855 (First Order, GLCM, GLDM, GLSZM, GLRLM, NGTDM, Wavelet) | Wilson test & Pearson correlation & LASSO (n=22) | LR | (a) AUC=0.91, (b) AUC=0.89 (internal validation) |
| (74) | Chiappa V. | Detection of benign and malignant ovarian masses | 241 US (monocentric) | n=319 (First Order, GLCM, GLDZM, GLSZM, GLRLM, NGTDM) | ICC & Nested ten-fold (n= 306) | SVM | AUC=0.89 , sens=0.81, spec=0.81, acc=0.81 (internal validation) |
| (75) | Yu X. | To discriminate between serous borderline ovarian and serous malignant ovarian tumours | 182 MRI (monocentric) | n=1167 (First Order, GLCM, GLRLM, GLSZM, NGTDM, GLDM) | ICC & LASSO (n=9) | SVM | AUC=0.86, acc=0.78, sens=0.80, spec=0.75 (internal validation) |
| (76) | Zhu H. | To differentiate epithelial ovarian cancer | 101 CT (monocentric) | n=148 (First Order GLCM, NGLDM, GLRLM, GLSZM) | Mann-Whitney U test & LASSO (n=8) | LR | sens=0.98, spec=0.67, AUC=0.78 (internal validation) |
| (77) | Meier A. | To detect the presence of BRCA mutations in ovarian cancer | 88 CT (monocentric) | n=7 (GLCM, intersite similarity matrix) | N.A. | Wilcoxon rank-sum tests | None of the evaluated texture metrics were able to assess BRCA mutation (p=0.05). |

Note: clear cell renal cell carcinoma (ccRCC), papillary renal cell carcinoma (pRCC), Computer Tomography (CT), Magnetic Resonance Imaging (MRI), gray-level co-occurrence (GLCM), gray-level run-length (GLRLM), gray-level dependence (GLDM), gray-level size zone (GLSZM), neighbouring gray tone difference (NGTDM), genetic algorithms (GA), maximum relevance minimum redundancy (MRMR), affinity propagation (AP), Intra-Class Correlation (ICC), least absolute shrinkage and selection operator (LASSO), Logistic Regression (LR), Support Vector Machines (SVM), Random Forest (RF), Decision Tree (DT), naïve Bayesian (NB), Neural Network (NN), Deep Learning (DL), Area Under the Curve (AUC), sensibility (sens), specificity (spec), accuracy (acc), Positive Predictive Value (PPV), Negative Predictive Value (NPV), Not Available (N.A.).

S-TABLE VIII: PAPERS RELATED TO CERVICAL AND ENDOMETRIAL CANCER

| Paper | First Author | Aims | Dataset | Features Extracted | Feature Selection | Classifier | Results  (best performing model) |
| --- | --- | --- | --- | --- | --- | --- | --- |
| (25) | Liu Y. | To predict normal-sized lymph node-metastasis in the cervice | 219 CT  (10 centres) | n=1409 (First Order, t GLCM, GLDM, GLRLM, NGTDM, GLSZM) | MRMR (n=564) | LR, SVM, DT, RF and NN | sens= 0.74, spec=0.80, acc=0.75, AUC=0.80 (external validation) |
| (15) | Veeraraghavan H. | (a) To detect  endometrial cancer genetic mutations  (b) To differentiate tumour mutational Burden‐High tumours | 150 CT (monocentric) | n=200 (First Order, GLCM, GLRLM, GLSZM, NGTDM, NGLDM + clinical variables) | MRMR & recursive RF  ((a) n=21; (b) n=12) | RF | (a): AUC=0.78. (b): AUC=0.87. (internal validation) |
| (80) | Fasmer K. E. | To assess endometrial cancer aggressiveness | 138 MRI (monocentric) | n=15 (First Order, GLCM, GLRLM) | LASSO & Ridge (elastic net) (n=12) | LR | AUC=0.63 (internal validation) |

Note: clear cell renal cell carcinoma (ccRCC), papillary renal cell carcinoma (pRCC), Computer Tomography (CT), Magnetic Resonance Imaging (MRI), gray-level co-occurrence (GLCM), gray-level run-length (GLRLM), gray-level dependence (GLDM), gray-level size zone (GLSZM), neighbouring gray tone difference (NGTDM), genetic algorithms (GA), maximum relevance minimum redundancy (MRMR), affinity propagation (AP), Intra-Class Correlation (ICC), least absolute shrinkage and selection operator (LASSO), Logistic Regression (LR), Support Vector Machines (SVM), Random Forest (RF), Decision Tree (DT), naïve Bayesian (NB), Neural Network (NN), Deep Learning (DL), Area Under the Curve (AUC), sensibility (sens), specificity (spec), accuracy (acc), Positive Predictive Value (PPV), Negative Predictive Value (NPV), Not Available (N.A.).

S-TABLE IX: PAPERS RELATED TO PROSTATIC CANCER

| Paper | First Author | Aims | Dataset | Features Extracted | Feature Selection | Classifier | Results  (best performing model) |
| --- | --- | --- | --- | --- | --- | --- | --- |
| (24) | Bagher-Ebadian H. | To detect prostatic cancer for the dose painting therapy | 127 MRI  (2 centres) | n=168 (First Order, GLCM, GLRLM, Laws, Wavelet, Gabor) | ANOVA (n=10) | NN | AUC=0.94, PPV=0.95, NPV=0.92  (external validation) |
| (10) | Nicoletti G. | To distinguish between high and low aggressive prostate cancer | 108 MRI  (2 centres) | n=290 (First Order, GLCM, GLDM, GLRLM, NGTDM, GLSZM) | MRMR, GA, AP (n=2) | SVM, DT, ensemble learning | AUC=0.81, sens=0.77, spec=0.93 (external validation) |
| (13) | Giannini V. | To assess prostate cancer aggressiveness | 131 MRI  (2 centres) | n=92 (First Order, GLCM, GLRLM) | AUC (n=6) | SVM | AUC=0.81 (external validation) |
| (14) | Qi Y. | To detect prostate cancer | 199 MRI (monocentric) | n=2104 (First Order, GLCM, GLDM, GLRLM, GLSZM, NGTDM, Wavelet, Laplacian + clinical variables) | Pearson chi-squared test (n=90) | LR | AUC=0.93, sens=0.81, spec=0.92  (internal validation) |
| (83) | Woźnicki P. | a) To detect malignant prostatic cancer  b) To categorise between malignant and benign prostatic lesions,  (c) To categorise between clinically significant insignificant prostate cancer | 191 MRI (monocentric) | n=367 (First Order, GLRLM, GLSZM, GLDM + clinical variables) | MRMR (n=15) | SVM, RF, LR, XGboost | a) AUC=0.89; (b) AUC=0.89, (c) AUC=0.84  (internal validation) |
| (84) | Chen T. | To assess prostate cancer aggressiveness | 182 MRI (monocentric) | n=396 (First Order, GLCM, GLRLM, form factor parameters) | ANOVA, Kruskal–Wallis test, univariate logistic, LASSO, Spearman test & RF (n=10) | LR | AUC=0.93 (internal validation) |
| (85) | Zhang L. | To assess prostate cancer aggressiveness | 139 MRI (monocentric) | n=788 (First Order, GLCM, GLDM, GLRLM, NGTDM, GLSZM, Wavelet) | Ranking method & LASSO (n=9) | LR | AUC=0.90 (internal validation) |
| (82) | Hu L. | To detect prostate cancer | 136 MRI (monocentric) | n=1016 (First Order, GLCM, GLDM, GLSZM, GLRLM, NGTDM, Wavelet, Laplacian + clinical variables) | Spearman correlation & MRMR & LASSO (n=12) | LR | AUC=0.93  (internal validation) |

Note: clear cell renal cell carcinoma (ccRCC), papillary renal cell carcinoma (pRCC), Computer Tomography (CT), Magnetic Resonance Imaging (MRI), gray-level co-occurrence (GLCM), gray-level run-length (GLRLM), gray-level dependence (GLDM), gray-level size zone (GLSZM), neighbouring gray tone difference (NGTDM), genetic algorithms (GA), maximum relevance minimum redundancy (MRMR), affinity propagation (AP), Intra-Class Correlation (ICC), least absolute shrinkage and selection operator (LASSO), Logistic Regression (LR), Support Vector Machines (SVM), Random Forest (RF), Decision Tree (DT), naïve Bayesian (NB), Neural Network (NN), Deep Learning (DL), Area Under the Curve (AUC), sensibility (sens), specificity (spec), accuracy (acc), Positive Predictive Value (PPV), Negative Predictive Value (NPV), Not Available (N.A.).
